# Risk factors for glucocorticoid induced osteoporosis in young adults

**DOI:** 10.3389/fendo.2025.1528962

**Published:** 2025-07-11

**Authors:** Helena Florez, Josep Lluis Carrasco, Martina Barberá, José Hernández-Rodríguez, Africa Muxi, Anastasia Mocritcaia, Sergio Prieto-González, Maria C. Cid, Ana Monegal, Núria Guañabens, Pilar Peris

**Affiliations:** ^1^ Metabolic Bone Diseases Unit. Department of Rheumatology. Hospital Clinic, University of Barcelona, Institut d’Investigacions Biomèdiques August Pi i Sunyer (IDIBAPS), Barcelona, Spain; ^2^ Biostatistics, Department of Basic Clinical Practice, University of Barcelona, Barcelona, Spain; ^3^ Vasculitis Research Unit, Department of Autoimmune Diseases, Hospital Clínic, University of Barcelona, Institut d’Investigacions Biomèdiques August Pi i Sunyer (IDIBAPS), Barcelona, Spain; ^4^ Department of Nuclear Medicine, Hospital Clinic, University of Barcelona, Barcelona, Spain

**Keywords:** glucocorticoid-induced osteoporosis, hypogonadism, young patients, fragility fracture, risk factors

## Abstract

**Introduction:**

Glucocorticoid-induced osteoporosis (GIOP) is one of the most frequent causes of secondary osteoporosis, especially in young subjects. However, current research and guidelines have scarcely addressed the therapeutic approach and risk factors for GIOP in adults less than 50 years of age. The aim of the study was to analyze if factors related to the development of glucocorticoid-induced osteoporosis (GIOP) and fragility fractures (FF) differ according to age.

**Methods:**

127 patients on chronic glucocorticoid (GC) treatment were analyzed, including GC doses and duration, disease activity, FF, anthropometric data, bone metabolism parameters (including sex steroids), bone mineral density, trabecular bone score, and radiologic vertebral fractures; defining GIOP as densitometric osteoporosis and/or FF. Young subjects (<50 years old) were compared with those ≥50 years for risk factors of GIOP and FF.

**Results:**

GIOP prevalence was similar in both age groups: <50 (n=36) 44.4% *vs.* 46.1% ≥50 years (n=91). Five subjects <50 (13.9%) and 30 ≥50 years (33%) presented FF (p=0.046). Having a higher body mass index (BMI), disease activity was a differential risk factor for FF in young subjects, whereas hypogonadism was a risk factor independent of age.

**Conclusions:**

More than 40% of young subjects on chronic GC therapy had GIOP. A higher BMI and disease activity and particularly, hypogonadism seem to be factors related to FF development in these subjects. Evaluation of these risk factors can improve the identification of young subjects at increased risk of fracture.

## 1 Introduction

Glucocorticoid-induced osteoporosis (GIOP) is one of the most frequent causes of secondary osteoporosis, especially in young subjects (1). However, despite being a common cause of osteoporosis in this population, current research and guidelines have scarcely addressed the therapeutic approach and risk factors for GIOP in young subjects (2). In this sense, although fragility fracture (FF) rates are higher in postmenopausal and in older populations than in premenopausal cohorts (3–5), premenopausal women may also present an increased risk of vertebral fractures (VF) (up to 29%) when treated with high doses of glucocorticoids (GC) (2), with similar numbers in young adult males (6). Additionally, young subjects may even less frequently receive preventive care for GIOP than the older population, who currently remain suboptimally treated (7). Theoretically, younger individuals have a lower deleterious effect of GC on bone due to a higher expected bone strength related to age and hormonal status. Nonetheless, nearly 21% of premenopausal women with systemic lupus erythematosus (SLE) treated with high doses of GC develop fragility VF, indicating the need to improve the identification of this high-risk population (8, 9). Thus, whether the risk factors for presenting GIOP and fractures differ depending on age is not well known. Most clinical guidelines have difficulties in addressing preventive therapeutic approaches in young subjects treated with GIOP, particularly in premenopausal women. Moreover, the FRAX index, the most widely used tool to estimate fracture risk, is only applicable for subjects ≥ 40 years, further hindering the evaluation of the younger population (10).

We recently evaluated the risk factors for fracture development in subjects with rheumatologic autoimmune disease chronically treated with GC (11). In this study, the presence of hypogonadism was the principal risk factor for fracture development, observing an additional effect of GC boluses. Moreover, in this cohort we observed that complementary evaluation of the trabecular bone score (TBS) showed a greater discriminative power than bone mineral density (BMD) for fracture risk assessment in GC-treated patients and a high negative predictive value for identifying GC-treated subjects at low risk of fracture (12).

The aim of this study was to evaluate whether the risk factors for presenting GIOP and fractures differ depending on age. Therefore, we analyzed the prevalence of GIOP and FF in long-term GC-treated patients and the risk factors related to their development according to age categorized as < and ≥50 years.

## 2 Materials and methods

### 2.1 Study design and participants

**Figure 1:**
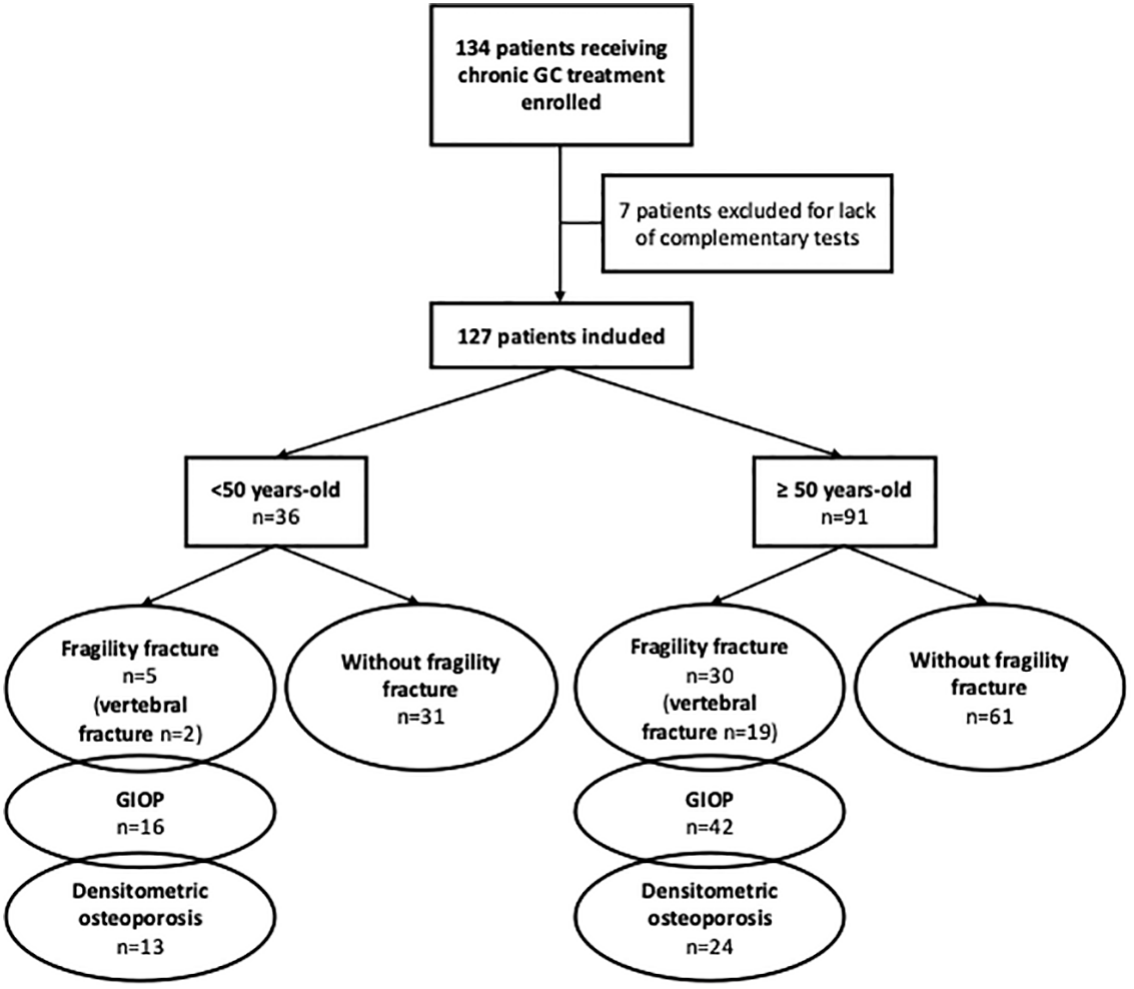
Distribution of patients in the different subgroups. GC, glucocorticoid; GIOP, glucocorticoid-induced osteoporosis (Fragility fracture and/or densitometric osteoporosis).

The study design has been previously published (11). Briefly, this was a cross-sectional study performed from August 2017 to April 2018 including 127 consecutive adult patients (aged >18 years) on chronic GC treatment (≥5 mg/day of prednisone or equivalent, for >3 months) for rheumatologic autoimmune disease referred to our Bone Metabolism Unit for osteoporosis assessment. Daily doses of glucocorticoids (GC) were given orally, and the two types of GC prescribed were prednisone or methylprednisolone (indistinctly, at the discretion of the treating physician). Intravenous GC boluses were of methylprednisolone. The cumulative GC dose was calculated taking into account the oral GC, intravenous bolus and infiltrations (triamcinolone acetonide) received, and is expressed in mg/day of prednisone or equivalent. Patients were evaluated according to age (< 50 vs. ≥ 50 years); this cut-off point of age was chosen because it is a criterion used in several GIOP guidelines to indicate preventive treatment of GIOP, and several studies have also described an increased risk for fracture over this age (13–16). The distribution of patients in the different subgroups is shown in **Figure 1**.

All patients provided written informed consent to participate, the study has been approved by the Ethics Committee of the Hospital Cliínic de Barcelona (July 26^th^, 2017; Reg. HCB/2017/0457) and has therefore been performed in accordance with the ethical standards laid down in the 1964 Declaration of Helsinki and its later amendments.

### 2.2 Assessments

Clinical evaluation included medical history focusing on osteoporosis risk factors: the presence of menopause (defined as the cessation of menstruation ≥12 months previously and considered as the presence of hypogonadism in women), history of all previous FF reported by the patients (considered as fractures resulting from low energy trauma, excluding fingers, toes, and skull), falls in the previous year, type, duration and activity of the autoimmune disease, GC doses and duration and the use of additional immunosuppressant agents. Anthropometric data (height, weight, body mass index [BMI] [kg/m^2^]) were also collected.

Blood samples were obtained between 8:00 and 10:00 a.m. after overnight fasting, and included acute phase reactants (erythrocyte sedimentation rate [ESR] and C-reactive protein [CRP]), serum creatinine, glomerular filtration rate (GFR), calcium, phosphate, bone turnover markers (BTM) including serum procollagen type I amino-terminal propeptide (PINP) and the cross-linked C-terminal telopeptide of type I collagen (CTX), parathyroid hormone (PTH) and 25-hydroxyvitamin D serum levels. Gonadotropins and total testosterone levels were determined in men. Hypogonadism in males was defined by testosterone levels <250 ng/dL (17). In women, menopause reported by the patient was considered as hypogonadism, and in doubtful cases of amenorrhoea/perimenopause gonadotropins and estrogen levels were tested.

DXA (Lunar Prodigy, General Electric Medical Systems, WI, USA) was used to assess BMD (g/cm^2^) of the lumbar spine and femur. Densitometric osteoporosis was defined according to the WHO criteria with T‐score values ≤‐2.5 (in subjects ≥50 years) or Z‐score values <‐2 (in subjects <50 years) (18–20).

The TBS was calculated using TBS iNsight^®^ software (version 3.0.2.0) (Medimaps group, Geneva, Switzerland) on the DXA lumbar spine images. A TBS value <1.230 was considered as degraded microarchitecture (DMA) (12).

Spinal X-rays were obtained to evaluate the presence of VF.

After clinical assessment and taking into account the clinical practice guidelines recommendations (10), antiosteoporotic treatment (including calcium, if dietary calcium was not sufficient, and vitamin D supplementation) was prescribed to the candidate patients.

### 2.3 Statistical analysis

Statistical analyses were performed using R v4.2.1 (21). Quantitative variables were described using means and standard deviations, or medians and quartiles in data not following a normal model. Qualitative variables were summarized using percentages and frequencies. We analyzed the risk factors for densitometric OP, FF or GIOP (FF and/or densitometric OP in the same variable) according to age groups of <50 and ≥50 years. The association with qualitative covariates was assessed using the chi-square and Fisher’s exact test when applicability conditions were not met. Adjusted odds ratios (OR) and p-values were obtained by applying logistic regression models. The association with quantitative covariates was assessed by comparing the means using t-tests. Linear models were used when adjustment for confounding covariates was needed. The difference between the risk factors in the groups <50 and ≥50 years was evaluated using the significance of the interaction in the model between the risk factor and the age group. Results were considered as significant if p <0.05 except for the case of multivariate model analysis where a p <0.1 was considered as sufficient to keep the covariates in the model.

## 3 Results

Of the total of 127 study patients on chronic GC treatment, 36 were <50 years (median age: 39 years). **Table 1** shows the clinical characteristics of the subjects according to age. The type of autoimmune disease differed depending on age: while SLE was predominant in younger patients, polymyalgia rheumatica, giant cell arteritis and inflammatory myopathies were more frequent in individuals over 50 years (**Supplementary Table 2**). Consequently, the use of immunosuppressant agents was more frequent in young subjects (77.8% *vs.* 42.9%, p=0.001), with methotrexate, hydroxychloroquine and azathioprine being the most frequently prescribed immunosuppressants in both group of patients. Although young subjects were receiving or had received more frequently immunosuppressive treatment, no significant differences were found in the development of osteoporosis and/or fractures. Patients <50 years received higher cumulative GC doses (8.9 *vs.* 6.4 g, p=0.02) with longer duration of GC treatment. The prevalence of GIOP was similar in both groups (44.4% in <50 years *vs.* 46.2% in ≥50 years), however, FF (14% *vs.* 33%, p=0.046) and VF (5.6% *vs.* 21.1%, p=0.036) were less frequent in young patients, as were the presence of degraded TBS values (<1.230; 8.3% *vs.* 69.2, p<0.001) (**Table 1**). In addition, no differences in the presence of FF were observed in relation to the duration of GC treatment (< 2 or ≥ 2 years of CG treatment) among groups (data not shown).

**Table 1:** Clinical characteristics of the patients according to age.

	< 50 years (n=36)	≥ 50 years (n=91)	P
Age (years, median, [range])	39 [31.3-45]	73 [64-79]	<0.001*
Gender (F/M, n)	27/9	53/38	0.12
BMI (Kg/m^2^, mean ± SD)	23.58± 4.7	27.97± 4.0	<0.001*
Menopause (in women, %)	5 (18.5)	53 (100)	<0.001*
Autoimmune disease duration (months, median, [range])	32.75 [9.9-122.1]	15.50 [4.8-53]	0.06
Current GC dose (prednisone or equivalent; mg/day, median, [range])	10 [5-20]	8.8 [5-15]	0.54
Cumulative GC dose (prednisone or equivalent; g, median, [range])	8.9 [5.4-26.8]	6.4 [2.5-13.1]	0.02*
GC treatment duration (months, median, [range])	32.8 [9.5-98.1]	14 [5.3-40]	0.02*
Intravenous GC boluses (methylprednisolone; n, %)	16 (44.4)	27 (29.7)	0.17
Immunosuppressant agents (n, %)	28 (77.8)	39 (42.9)	0.001*
Patients with any fragility fracture (n, %)	5 (13.9)	30 (33)	0.046*
Patients with vertebral fracture (n, %)	2 (5.6)	19 (21.1)	0.036*
Densitometric osteoporosis (n, %)	13 (36.1)	24 (26.4)	0.38
Degraded microarchitecture (n, %)	3 (8.3)	63 (69.2)	<0.001*
GIOP (densitometric OP and/or FF) (n, %)	16 (44.4)	42 (46.2)	1.00
Creatinine (mg/dL, median, [range])	0.72 [0.6-0.8]	0.82 [0.73-1.02]	<0.001*
GFR (mL/min, median, [range])	90 [90-90]	81 [60.5-89.5]	<0.001*
CRP (mg/dL, median, [range])	0.08 [0.02-0.49]	0.40 [0.14-0.93]	0.001*
ESR (mm/h, median, [range])	9 [6.8-21.3]	16 [7.5-28]	0.11
25-hydroxyvitamin D (ng/mL, median, [range])	25.8 [19.2-28.9]	25.2 [18.3-32.5]	0.85
PINP (ng/mL, median, [range])	42.8 [26.6-59.8]	21.9 [15.3-33.9]	<0.01*^&^
CTX (ng/mL, median, [range])	0.36 [0.24-0.51]	0.28 [0.15-0.45]	0.79^&^
Hypogonadism (men + women) (n, %)	5 (13.9)	61 (67.03)	<0.001*

F, female; M, male; BMI, body mass index; SD, standard deviation; GC, glucocorticoid; GIOP, glucocorticoid-induced osteoporosis; OP, osteoporosis; FF, fragility fracture; GFR, glomerular filtration rate; CRP, C-reactive protein; ESR, erythrocyte sedimentation rate; PINP, procollagen type I N-terminal propeptide; CTX, C-terminal telopeptide of type I collagen.

&: P-values adjusted for antiosteoporotic treatment.

Statistically significant results are marked with *.

As expected, the presence of menopause (100% *vs.* 18.5%, p<0.001) and hypogonadism (including men and women) (67% *vs.* 13.9%, p<0.001) was more frequent in patients ≥50 years. On the other hand, the BMI was higher in the older group of patients (28.0 *vs.* 23.6, p<0.001) (**Table 1**). Concerning biochemical parameters, younger patients showed higher BTM values, particularly PINP serum levels (even when adjusted for antiosteoporotic treatment), whereas the group ≥50 years presented a lower GFR (81 *vs.* 90 ml/min, p<0.001) and higher CRP and PTH values ​​(0.40 *vs.* 0.08 mg/dL, p=0.001; 66 *vs.* 48 pg/dL, p<0.001, respectively). At the time of assessment, 13.9% of patients <50 years of age were receiving or had received antiosteoporotic treatment *vs.* 52.8% of patients ≥ 50 years old (p<0.001), which increased to up to 75% of patients after our clinical assessment.

On comparing younger patients (<50) with and without GIOP, as expected, those with GIOP had significantly lower BMD and TBS values (**Table 2**). Compared to young non-GIOP patients, 25% of young GIOP subjects (all women) presented associated hypogonadism (**Table 2**). Five young patients presented FF, 2 being VF (other fracture locations were radius, tibia, pelvis, metatarsus, and cuboid bone). Although in our sample there was a higher prevalence of hypogonadism among young subjects with fractures (40% *vs.* 9.7%), no significant differences were observed between these groups of patients (young subjects with and without FF) (**Table 3**).

**Table 2:** Clinical characteristics of patients with and without GIOP according to age.

< 50 YEARS (n=36)	With GIOP (n=16)	Without GIOP (n=20)	P
Age (years, median, [range])	40 [31.3-45.5]	37 [31.3-44.3]	0.54
Gender (F/M, n)	12/4	15/5	1.00
BMI (Kg/m^2^, mean ± SD)	24.4 ± 5.2	22.9 ± 4.3	0.37
Menopause (%)	4 (33.3%)	1 (6.7%)	0.14
Current GC dose (prednisone or equivalent; mg/day; median, [range])	10 [6.9-15.6]	10 [5-20]	0.82
GC cumulative dose (prednisone or equivalent; g; median, [range])	9.5 [7.4-21.4]	8.8 [4.1-26.8]	0.46
GC treatment duration (months; median, [range])	20.5 [9.5-125.6]	41.3 [11-81.4]	0.90
Hypogonadism (men + women) (n, %)	4 (25)	1 (5)	0.15
PINP (ng/mL; mean ± SD)	45.1 ± 16.2	46.1 ± 25.3	0.88
CTX (ng/mL; mean ± SD)	0.38 ± 0.18	0.40 ± 0.2	0.79
CRP (mg/dL, median, [range])	0.06 [0.03-0.56]	0.09 [0.02-0.41]	0.79
ESR (mm/h, median, [range])	7 [6-16.25]	9 [8-27.8]	0.12
Lumbar spine T-score (mean ± SD)	-2.01 ± 0.84	-0.07 ± 1.29	<0.001*
Femoral neck T-score (mean ± SD)	-1.59 ± 1.09	-0.66 ± 1.32	0.03*
Total hip T-score (mean ± SD)	-1.66 ± 0.94	-0.49 ± 1.26	0.004*
TBS (mean ± SD)	1.321 ± 0.090	1.394 ± 0.105	0.03*
Degraded microarchitecture (n, %)	2 (12.5)	1 (5)	0.33

GIOP, Glucocorticoid-induced osteoporosis; F, female; M, male; BMI, body mass index; SD, standard deviation; GC, glucocorticoid; PINP, procollagen type I N-terminal propeptide; CTX, C-terminal telopeptide of type I collagen; CRP, C-reactive protein; ESR, erythrocyte sedimentation rate; TBS, trabecular bone score.

Statistically significant results are marked with *.

**Table 3:** Clinical characteristics of young patients (<50 years) with and without fragility fractures.

	With fragility fracture (n=5)	Without fragility fracture (n=31)	P
Age (years, median, [range])	45 [40-47]	37 [29-44]	0.08
Gender (F/M, n)	4/1	23/8	1
BMI (Kg/m^2^, mean ± SD)	25.4 ± 2.3	23.3 ± 4.9	0.15
Menopause (in women, %)	2 (50)	3 (13)	0.14
GC cumulative dose (prednisone or equivalent; g; median, [range])	8.2 [7.6-19.3]	8.9 [4.4-27.0]	0.54
GC treatment duration (months; median, [range])	23 [11.5-142.5]	33.5 [9.5-86.8]	0.78
Hypogonadism (men + women) (n, %)	2 (40)	3 (9.7)	0.13
PINP (ng/mL; median, [range])	38.3 (8.7)	46.9 (22.7)	0.15
CTX (ng/mL; median, [range])	0.37 (0.20)	0.39 (0.19)	0.80
CRP (mg/dL, median, [range])	0.9 [0.4-1.0]	0.06 [0.02-0.3]	0.06
ESR (mm/h, median, [range])	10 [7-17]	9 [6-23.5]	0.91
Lumbar spine T-score (mean ± SD)	-1.26 ± 0.63	-0.91 ± 1.57	0.40
Femoral neck T-score (mean ± SD)	-1.36 ± 0.64	-1.04 ± 1.38	0.42
Total hip T-score (mean ± SD)	-1.24 ± 0.67	-0.99 ± 1.34	0.53
Densitometric osteoporosis (n, %)	2 (40)	11 (35.5)	1
TBS (mean ± SD)	1.370 ± 0.1	1.320 ± 0.1	0.41
Degraded microarchitecture (n, %)	1 (20)	2 (6.5)	0.55

F, female; M, male; BMI, body mass index; SD, standard deviation; GC, glucocorticoid; PINP, procollagen type I N-terminal propeptide; CTX, C-terminal telopeptide of type I collagen; CRP, C-reactive protein; ESR, erythrocyte sedimentation rate; TBS, trabecular bone score.

In the multivariate analysis, when comparing patients with FF according to age (**Supplementary Table 1**; [in this table several values were transformed to logarithmic scale for the analysis and adjusted for age and BMI]), young subjects (< 50 years) had a higher BMI and CRP values compared to those without fractures (29.6 ± 1.3 *vs.* 26.95 ± 0.6, p=0.048 and -0.87 ± 0.7 *vs.* -2.51 ± 0.3 mg/dL, p=0.03; respectively), whereas in subjects over 50 years old with fractures/FF, the BMI and the T-score at lumbar spine were lower and these subjects had received higher cumulative GC doses than patients with no-fractures (28.3 ± 0.5 *vs.* 29.9 ± 0.4, p=0.02; -1.08 ± 0.27 *vs.* -0.06 ± 0.21, p=0.003; 9.1 ± 0.2 *vs.* 8.6 ± 0.2 g, p=0.03, respectively). Of interest, despite the BMI being significantly higher in subjects ≥ 50 than in those <50 years (**Table 1**), this factor had a differential effect in young patients, as young subjects with FF had a higher BMI than those without FF.

Moreover, the presence of hypogonadism was a notable risk factor for FF independently of age (OR 4.89; 95%CI 1.36-17.59, p=0.02) (**Supplementary Table 1**) and also for GIOP in all patients (OR 3.51, 95% CI 1.18-10.46, p=0.02) and in those ≥50 years (OR 4.22, 95% CI 1.06-16.75, p=0.04).

## 4 Discussion

This study confirms the high prevalence of GIOP in young subjects receiving chronic GC treatment and reinforces the need to evaluate the presence of osteoporosis in all GC-treated patients, independently of age. However, the development of FF clearly differed according to age, being less frequent in individuals under 50. This difference was even more pronounced in the case of VF, highlighting the importance of the evaluation of risk factors for fracture according to patient age.

In this sense, 44.4% of the younger patients had GIOP, with 14% presenting FF. While young subjects showed a lower prevalence of fractures, the presence of densitometric osteoporosis was similar to that in older patients (36.1% in <50 *vs.* 26.4% in ≥50), indicating the need to evaluate other components of bone strength in these subjects. Recent studies have shown that TBS may have a greater discriminative power than BMD for fracture risk assessment in GC-treated patients (12, 22, 23). We found that the presence of degraded TBS values markedly differed according to age: with 69.2% of patients over 50 showing degraded TBS values compared to 8.3% of the younger subjects (one with VF). The low prevalence of degraded TBS values in young subjects coincided with a low prevalence of fractures in this population, confirming the high negative predictive value of TBS for fractures that we have previously reported in the same cohort of patients as that included in the present study (12)). This suggests that TBS could be useful for evaluating subjects at risk of fracture. Nevertheless, whether or not this predictive value differs depending on age is not known. Clearly, further studies analyzing this finding are recommended.

The doses and duration of GC treatment are well known factors related to the development of fractures in GC-treated patients, with a dose-dependent increase in fracture risk (10, 24). In our study, younger patients received higher cumulative doses of GC than subjects >50, with a similar mean GC dose per day at the time of evaluation. Despite these higher cumulative GC doses, young subjects presented a lower incidence of fractures, even when these subjects were evaluated according to GC treatment duration (< 2 *vs.* ≥ 2 years: a period of time that has been associated with increased risk fractures) (25), again indicating that in younger subjects other factors, apart from GC doses and duration, need to be considered.

Our group and others have shown that hypogonadism is an important factor for the development of fractures in both men and women (11). In this study, hypogonadism was again the principal factor related to fracture development in both <50 and >50-year-old subjects, further reaffirming the relevance of this factor. Whether other factors that have been associated with a higher bone loss during the perimenopausal period, such as increased follicle stimulating hormone levels, may have influenced this bone loss in these subjects is not known (26). In addition, whether hormone replacement therapy can be recommended in all patients with GIOP and hypogonadism remains unclear. Nonetheless, it could be considered in combination with antiosteoporotic therapy in hypogonadal patients who are symptomatic, provided there are no contraindications, since there is insufficient evidence to support hormone replacement therapy alone for GIOP treatment (27).

Of note, in the multivariate analysis, hypogonadism, increased BMI, and inflammatory disease activity were related to GIOP and fractures in young subjects, which may be factors for identifying young subjects at risk of fracture. We believe it is important to consider these findings for future research.

Several studies have described an increased risk of VF in premenopausal women with increasing age receiving high GC doses, particularly in those over 40 (10, 28). In the present study, young subjects with FF tended to be older, with a mean age of 45, and only one out the 5 young subjects with fractures was under 40 years old; nevertheless this difference did not reach statistical significance.

Persistent inflammation is related to bone loss in autoimmune diseases (29–31). Nevertheless, whether the type of rheumatologic autoimmune disease plays an additional role in bone loss in these patients is not well known, with the exception of rheumatoid arthritis, which is a known independent risk factor for bone loss and fractures, regardless of GC exposure (29–32). Interestingly, pro-inflammatory factors resulting from systemic inflammation, such as IL-6, IL-1β or TNF, can promote bone resorption by acting directly on bone cells. However, these factors can simultaneously stimulate the upregulation of 11β-HSD1, the GC-activating enzyme, and the resulting increase in cortisol, inflammation would reduce inflammation-related bone loss (30, 33). Several studies have indicated the importance of controlling inflammation as an essential part of the therapeutic approach in these subjects (29). Thus, GC treatment may reduce underlying inflammatory activity, thereby mitigating the deleterious effects of bone loss, but also exerts an opposite effect, acting directly on bone cells inducing GIOP (30, 33). In our series, we did not observe a relationship with the type of disease and the presence of GIOP. However, there were several different autoimmune diseases making comparisons unfeasible and only 2 patients had rheumatoid arthritis. On the other hand, CRP values (a marker of disease activity) were found to be a risk factor for fracture in young subjects. Conversely, we also observed that young subjects presented significantly higher BTM values, particularly of PINP, than subjects over 50 years. Although the causes of these differences are not well known, the more frequent use of antiosteoporotic treatment in adult subjects could partly explain these results. Thus, after adjusting BTM for age and antiosteoporotic treatment, these differences were no longer significant for CTX, while PINP values remained significant after adjustment. Whether or not other factors, such as disease activity, comorbidities, other medications and/or age itself could contribute to this finding is not known.

Interestingly, young subjects with fractures presented a higher BMI than those without fractures, making BMI a differential risk factor for fracture when comparing patients <50 vs. ≥50 years, with only young subjects presenting a high risk of fracture associated with increased BMI. This finding could be related to higher GC exposure in young people, either with higher and prolonged doses or because of increased intracellular availability at the tissue level. Previous studies have also observed higher BMI values and hyperlipidemia in fractured subjects treated with GC (34), reflecting the prejudicial impact of prolonged exposure to GC on body composition (central obesity) in some individuals (35, 36). The present data suggest the need to better evaluate the role of this risk factor in young GC-treated individuals since it may identify a high-risk group for fracture.

Our study has several limitations, such as those related to the cross-sectional nature of the study. Additionally, the low prevalence of fractures in young individuals and the sample size of this subgroup of patients could also constitute a partial limitation in the analysis, since the power of the study with this sample was low (43.4%) to detect a medium effect size of association. Nevertheless, although the power is low, thereby indicating that we could have missed other associations, this finding reinforces our results, since only strong associations can be obtained with this statistical power. The different patterns of diseases, with longer disease duration and higher cumulative GC doses observed in young subjects could also be a limitation, as well as the higher proportion of women in the sample. However, it should be noted that these findings are inherent to the particular characteristics of rheumatologic autoimmune diseases, which differ according to age and sex. Moreover, despite having higher cumulative GC doses and a longer duration of the disease, and a similar prevalence of densitometric osteoporosis, young patients presented a much lower prevalence of FF, further indicating the need to identify this high-risk patient group. Nonetheless, the strengths of this study include the homogeneity of the characteristics of the patients (all on chronic GC treatment with doses ≥5 mg/day for an autoimmune disease), together with in-depth clinical evaluation, extensive bone metabolism analysis and radiological and DXA studies related to the development of fractures.

In conclusion, the prevalence of GIOP in subjects on chronic GC treatment is high (>40%) and independent of the age group (<50 and ≥50 years). However, FF are less frequent in young patients. The presence of hypogonadism is a determining risk factor for developing fractures, regardless of the age of the patient, indicating the need to implement preventive measures for the development of fractures in this type of patients. TBS evaluation in young subjects could be a useful complementary tool to identify those at risk, although further studies are needed to confirm its utility in this regard.

## Data Availability

The original contributions presented in the study are included in the article/**Supplementary Material**. Further inquiries can be directed to the corresponding author.
