# Supplementary material for: Risk factors for glucocorticoid induced osteoporosis in young adults
Source: Front Endocrinol (Lausanne). 2025 Jul 11;16:1528962. doi: 10.3389/fendo.2025.1528962 (PMC12289500; doi:10.3389/fendo.2025.1528962)
Supplement: Supplementary file 1 [file DataSheet1.pdf]

Supplementary material

Supplementary Table 1. Comparative analysis of risk factors for fragility fracture according to age.

FRAGILITY FRACTURE							
	< 50 years			≥ 50 years			Fracture–age group interaction
	With fracture	Without fracture	p	With fracture	Without fracture	P	p
**BMI** (Kg/m^2, mean ± SD)	29.6±1.3	26.9±0.6	0.048*	28.3±0.5	29.9±0.4	0.016*	0.005*
**Autoimmune disease duration** (months, mean ± SD#)	3.5±0.8	3.5±0.3	0.98	3.0±0.3	2.7±0.2	0.48	0.78
**Current GC dose** (prednisone or equivalent; mg/day, mean ± SD#)	2.1±0.3	2.4±0.2	0.41	2.30±0.1	2.3±0.1	0.98	0.46
**GC cumulative dose** (prednisone or equivalent; mg, mean ± SD#)	9.8±0.5	9.3±0.2	0.41	9.11±0.2	8.6±0.2	0.03*	0.85
**GC treatment duration** (months, mean ± SD#)	3.7±0.7	3.6±0.3	0.87	3.0±0.3	2.7±0.2	0.4	0.83
**CRP** (mg/dL, mean ± SD#)	-0.87±0.7	-2.51±0.3	0.026*	-1.14±0.3	-0.97±0.2	0.64	0.03*
**ESR** (mm/h, mean ± SD#)	2.55±0.37	2.64±0.17	0.81	2.78±0.15	2.83±0.11	0.79	0.92
**PINP** (ng/mL, mean ± SD#)	3.6±0.3	3.7±0.1	0.72	3.1±0.1	3.2±0.1	0.61	0.92
**CTX** (ng/mL, mean ± SD#)	-1.2±0.3	-1.15±0.1	0.85	-1.4±0.1	-1.35±0.1	0.7	1.00
**Lumbar spine T-score** (mean ± SD)	-0.001±0.68	0.14±0.31	0.84	-1.08±0.27	-0.06±0.2	0.003*	0.27

**Femoral neck T-score** (mean ± SD)	-1.24±0.46	-0.93±0.21	0.52	-1.61±0.19	-1.39±0.14	0.34	0.87
**Total hip T-score** (mean ± SD)	-0.97±0.49	-0.76±0.23	0.68	-1.24±0.20	-0.96±0.15	0.25	0.90
**TBS** (mean ± SD)	1.275±0.066	1.325±0.030	0.47	1.116±0.026	1.155±0.020	0.23	0.90
		**< 50 years**		**≥ 50 years**		**p**	
		**OR (95% CI)**	**p**	**OR (95% CI)**	**p**	**OR (95% CI)**	**p**
**Hypogonadism (men + women)** (n, %)		7.69 (0.85-69.58)	0.07	4.20 (0.87-20.33)	0.07	4.89 (1.36-17.59)	0.02*
**Intravenous GC boluses** (n, %)		0.69 (0.10-4.83)	0.70	2.11 (0.80-5.60)	0.13	1.79 (0.74-4.33)	0.20
**Immunosuppressive treatment** (n, %)		0.95 (0.18 - 5.06)	0.95	0.69 (0.25 – 1.90)	0.47	1.38 (0.20 – 9.67)	0.75
**Diabetes mellitus** (n, %)		0.86 (0.28 – 2.62)	0.79	0.94 (0.37 – 2.41)	0.90	0.91 (0.37 – 2.22)	0.22

Values adjusted for age and BMI
Variables transformed to logarithmic scale

BMI: body mass index, SD: standard deviation, GC: glucocorticoid, CRP: C-reactive protein, ESR: erythrocyte sedimentation rate, PINP: procollagen type I N-terminal propeptide, CTX: C-terminal telopeptide of type I collagen, TBS: trabecular bone score, OR: Odds ratio, CI: confidence interval

Supplementary Table 2. Types of autoimmune diseases according to age.

AUTOIMMUNE DISEASE	<50 years-old	≥ 50 years-old	Total
**Systemic vasculitis**	**11**	**44**	**55**
• Giant cell arteritis	0	26	26
• Eosinophilic granulomatosis with polyangiitis	2	5	7
• Panarteritis nodosa	3	3	6
• Granulomatosis with polyangiitis			
**Polymyalgia rheumatica**	**0**	**24**	**24**
**Systemic lupus erythematosus**	**10**	**3**	**13**
**Dermatomyositis**	**3**	**6**	**9**
**Others**	**11**	**10**	**21**
• Inflammatory myopathies	1	4	5
• Autoinflammatory syndromes	2	1	3
• Systemic sclerosis	1	1	2
• Rheumatoid arthritis			
